# A high-stringency blueprint of the human proteome

**DOI:** 10.1038/s41467-020-19045-9

**Published:** 2020-10-16

**Authors:** Subash Adhikari, Edouard C. Nice, Eric W. Deutsch, Lydie Lane, Gilbert S. Omenn, Stephen R. Pennington, Young-Ki Paik, Christopher M. Overall, Fernando J. Corrales, Ileana M. Cristea, Jennifer E. Van Eyk, Mathias Uhlén, Cecilia Lindskog, Daniel W. Chan, Amos Bairoch, James C. Waddington, Joshua L. Justice, Joshua LaBaer, Henry Rodriguez, Fuchu He, Markus Kostrzewa, Peipei Ping, Rebekah L. Gundry, Peter Stewart, Sanjeeva Srivastava, Sudhir Srivastava, Fabio C. S. Nogueira, Gilberto B. Domont, Yves Vandenbrouck, Maggie P. Y. Lam, Sara Wennersten, Juan Antonio Vizcaino, Marc Wilkins, Jochen M. Schwenk, Emma Lundberg, Nuno Bandeira, Gyorgy Marko-Varga, Susan T. Weintraub, Charles Pineau, Ulrike Kusebauch, Robert L. Moritz, Seong Beom Ahn, Magnus Palmblad, Michael P. Snyder, Ruedi Aebersold, Mark S. Baker

**Affiliations:** 1 Faculty of Medicine, Health and Human Sciences, Department of Biomedical Sciences, Macquarie University, North Ryde, NSW 2109 Australia; 2 Faculty of Medicine, Nursing and Health Sciences, Department of Biochemistry and Molecular Biology, Monash University, Melbourne, VIC 3800 Australia; 3 Institute for Systems Biology, 401 Terry Avenue North, Seattle, WA 98109 USA; 4 Faculty of Medicine, SIB-Swiss Institute of Bioinformatics and Department of Microbiology and Molecular Medicine, University of Geneva, CMU, Michel-Servet 1, 1211 Geneva, Switzerland; 5 Department of Computational Medicine and Bioinformatics, University of Michigan, Ann Arbor, MI 48109-2218 USA; 6 UCD Conway Institute of Biomolecular and Biomedical Research, School of Medicine, University College Dublin, Dublin, Ireland; 7 Yonsei Proteome Research Center, 50 Yonsei-ro, Sudaemoon-ku, Seoul, 120-749 South Korea; 8 Faculty of Dentistry, University of British Columbia, Vancouver, BC Canada; 9 Functional Proteomics Laboratory, Centro Nacional de Biotecnología-CSIC, Proteored-ISCIII, 28049 Madrid, Spain; 10 Department of Molecular Biology, Princeton University, Princeton, NJ 08544 USA; 11 Cedars Sinai Medical Center, Advanced Clinical Biosystems Research Institute, The Smidt Heart Institute, Los Angeles, CA 90048 USA; 12 Science for Life Laboratory, School of Engineering Sciences in Chemistry, Biotechnology and Health, KTH Royal Institute of Technology, 17121 Solna, Sweden; 13 Rudbeck Laboratory, Department of Immunology, Genetics and Pathology, Uppsala University, 75185 Uppsala, Sweden; 14 Department of Pathology and Oncology, Johns Hopkins University School of Medicine, Baltimore, MD 21224 USA; 15 Biodesign Institute, Arizona State University, Tempe, AZ USA; 16 Office of Cancer Clinical Proteomics Research, National Cancer Institute, NIH, Bethesda, MD 20892 USA; 17 State Key Laboratory of Proteomics, Beijing Proteome Research Center, National Center for Protein Sciences (Beijing), Beijing Institute of Lifeomics, Beijing, 102206 China; 18 Bruker Daltonik GmbH, Microbiology and Diagnostics, Fahrenheitstrasse, 428359 Bremen, Germany; 19 Cardiac Proteomics and Signaling Laboratory, Department of Physiology, David Geffen School of Medicine, University of California Los Angeles, Los Angeles, CA USA; 20 CardiOmics Program, Center for Heart and Vascular Research, Division of Cardiovascular Medicine and Department of Cellular and Integrative Physiology, University of Nebraska Medical Center, Omaha, NE 68198 USA; 21 Department of Chemical Pathology, Royal Prince Alfred Hospital, Camperdown, NSW Australia; 22 Indian Institute of Technology Bombay, Powai, Mumbai 400076 India; 23 Cancer Biomarkers Research Branch, National Cancer Institute, National Institutes of Health, 9609 Medical Center Drive, Suite 5E136, Rockville, MD 20852 USA; 24 Proteomics Unit and Laboratory of Proteomics, Institute of Chemistry, Federal University of Rio de Janeiro, Av Athos da Silveria Ramos, 149, 21941-909 Rio de Janeiro, RJ Brazil; 25 University of Grenoble Alpes, Inserm, CEA, IRIG-BGE, U1038, 38000 Grenoble, France; 26 Departments of Medicine-Cardiology and Biochemistry and Molecular Genetics, University of Colorado, Anschutz Medical Campus, Aurora, CO USA; 27 Consortium for Fibrosis Research and Translation, University of Colorado, Anschutz Medical Campus, Aurora, CO USA; 28 Division of Cardiology, Department of Medicine, University of Colorado, Anschutz Medical Campus, Aurora, CO USA; 29 European Molecular Biology Laboratory, European Bioinformatics Institute, Wellcome Trust Genome Campus, Hinxton, Cambridge, CB10 1SD UK; 30 School of Biotechnology and Biomolecular Sciences, University of New South Wales, Sydney, NSW Australia; 31 Department of Computer Science and Engineering, University of California, San Diego, 9500 Gilman Drive, Mail Code 0404, La Jolla, CA 92093-0404 USA; 32 Department of Biomedical Engineering, Lund University, Lund, Sweden; 33 Department of Biochemistry and Structural Biology, University of Texas Health Science Center San Antonio, UT Health, 7703 Floyd Curl Drive, San Antonio, TX 78229-3900 USA; 34 University of Rennes, Inserm, EHESP, IREST, UMR_S 1085, F-35042 Rennes, France; 35 Leiden University Medical Center, Leiden, 2333 The Netherlands; 36 Department of Genetics, Stanford School of Medicine, Stanford, CA 94305 USA; 37 Faculty of Science, University of Zurich, Zurich, Switzerland

**Keywords:** Proteomics, Proteomic analysis, Molecular medicine

## Abstract

The Human Proteome Organization (HUPO) launched the Human Proteome Project (HPP) in 2010, creating an international framework for global collaboration, data sharing, quality assurance and enhancing accurate annotation of the genome-encoded proteome. During the subsequent decade, the HPP established collaborations, developed guidelines and metrics, and undertook reanalysis of previously deposited community data, continuously increasing the coverage of the human proteome. On the occasion of the HPP’s tenth anniversary, we here report a 90.4% complete high-stringency human proteome blueprint. This knowledge is essential for discerning molecular processes in health and disease, as we demonstrate by highlighting potential roles the human proteome plays in our understanding, diagnosis and treatment of cancers, cardiovascular and infectious diseases.

## Abstract (web summary)

The Human Proteome Project (HPP) was launched in 2010 to enhance accurate annotation of the genome-encoded proteome. Ten years later, the HPP releases its first blueprint of the human proteome, annotating 90% of all known proteins at high-stringency and discussing the implications of proteomics for precision medicine.

## Introduction

A decade after the release of the draft Human Genome Project (HGP), the Human Proteome Organization (HUPO) leveraged this genomic encyclopedia to launch a visionary international scientific collaboration called the Human Proteome Project (HPP)^1–4^. Utilizing substantial community data, the HPP connects scientists, clinicians, industry, institutions and knowledgebase (KB) partners to create a framework for collaboration, data sharing and quality assurance—all targeted at discovering credible evidence for the entire complement of human genome-coded proteins (Box 1).

Here we report and discuss HUPO’s first high-stringency HPP blueprint (https://www.nextprot.org/about/statistics, data release 17-01-2020). This blueprint was assembled over 10 years by the HPP and covers >90% of the human proteome, paralleling progress made by the HGP^5^. This effort relied heavily upon community efforts that enabled HPP data inspection and re-analysis, culminating in the creation of a high-stringency human proteome KB. To illustrate the many historical innovations driving growth in proteomics, HUPO has created a historical timeline that will be released coincidentally with this publication (https://hupo.org/Proteomics-Timeline).

Box 1 **HPP decadal achievements**

- Generated a framework, plan and governance structure for community-based mapping of the human proteome.
- Confirmed neXtProt as the HPP reference knowledgebase and supported creation and use of ProteomXchange (PX) to register and make proteomics raw mass spectrometry (MS) and metadata available and reusable under FAIR (findable, accessible, interoperable and reusable) principles.
- Engaged neXtProt, PeptideAtlas, PRIDE and MassIVE as partners in the generation of annual neXtProt HPP release and a high-stringency HPP knowledgebase (KB).
- Encouraged community support of high-stringency protein inference and proteomic data analysis.
- Built MS data interpretation guidelines that promoted the application of standardized analysis of community human MS and proteomic data to progressively complete the human proteome parts list.
- Aligned with the Human Protein Atlasʼ (HPA) cell and tissue spatio-temporal maps in health/disease and supported community efforts to raise awareness of antibody specificity and quality assurance issues.
- Partnered with SRMAtlas to develop quantitative targeted proteomics assays for the analysis of key proteins and hallmark pathways/networks.
- Proposed and built global collaborative initiatives to investigate the biology of human health and disease at a proteomic-wide scale.
- Raised the profile and visibility of proteomics, as an essential component of life sciences and biomedical research by promoting the development of instrumentation and methods for proteoform analysis, as well as activity/function that cannot be addressed by genomics.
- Initiated a programme to determine the biological function for uncharacterized PE1 proteins (see below) that currently lack functional annotation.
- Established a HUPO Early Career Researcher network to engage, mentor and highlight research from young scientists/clinicians, while actively promoting gender and regional balance.

**Future goals**

- Establish a community initiative to systematically map all human proteoforms.
- Establish optimized workflows for human proteome detection, quantification and functional characterization, including low abundance and/or temporo-spatially restricted proteins.
- Continue to support and promote the provision of technical standards, metrics and stringent guidelines for confident protein identification and quantification.
- Create a comprehensive, accurate, publicly-accessible, reference human proteome knowledgebase, reusable under FAIR principles.
- Maintain education and training programmes in all aspects of proteomics including proteomic data analysis for early career researchers and clinical scientists.
- Be a focal point for life sciences researchers, pathologists, clinicians and industry communities seeking to translate and leverage proteomic and proteogenomic data to improve human health through: (i) greater understanding of the molecular mechanisms of common and rare diseases, (ii) identification of pathophysiological changes to generate disease and wellness diagnostic biomarkers, and (iii) development of new effective and safe personalized therapeutics.

## HPP mission and strategic aims

The HPP mission is to assemble and analyse community data, bringing increased granularity to our molecular understanding of the dynamic nature of the proteome, its modifications and relationships to human biology and disease. This aligns closely with HUPO’s aim of ‘translating the code of life’, providing crucial biochemical and cell biological information that genomics per se cannot deliver, while laying better foundations for diagnostic, prognostic, therapeutic and precision medicine applications.

From its inception, the HPP stated two strategic objectives as follows:

1. To credibly catalogue the human proteome parts list and discover its complexity (including posttranslational modifications (PTMs), splice variants, interactions and functions) by:
  - Establishing agreed, stringent, reliable standards,
  - Identifying >1 protein product from each protein-coding gene and
  - Detecting expression of the remaining missing proteins (see below).
2. To make proteomics an integrated component of multi-omics studies to advance life sciences, biomedical sciences and precision medicine.

Comparisons with the HGP are numerous. Both global projects are ambitious cooperative community efforts seeking to identify how genes (HGP) or proteins (HPP) help define the molecular mechanisms underlying health and disease. Both groups have implemented exhaustive data sharing and stringent quality control efforts. However, we now know that sequencing human genomes is necessary but not sufficient to understand the complexity of human biology or pathology. Knowledge of expressed proteins (including concentration, spatio-temporal localization, activities, protease-processed forms, transport, interactions, splice isoforms, PTMs and the many proteoforms derived from the proteome) cannot be predicted by genome sequencing alone.

## HPP structure and achievements

HUPO launched the HPP in 2010 at the 9th HUPO Annual World Congress in Sydney, with Gil Omenn as the inaugural chair. The HPP started without long-term funding. Financial support over the decade came through individual principal investigator projects, institutional infrastructure/core facilities and philanthropy, without large-scale, long-term, integrated multi-government strategic funding.

**Fig. 1:**
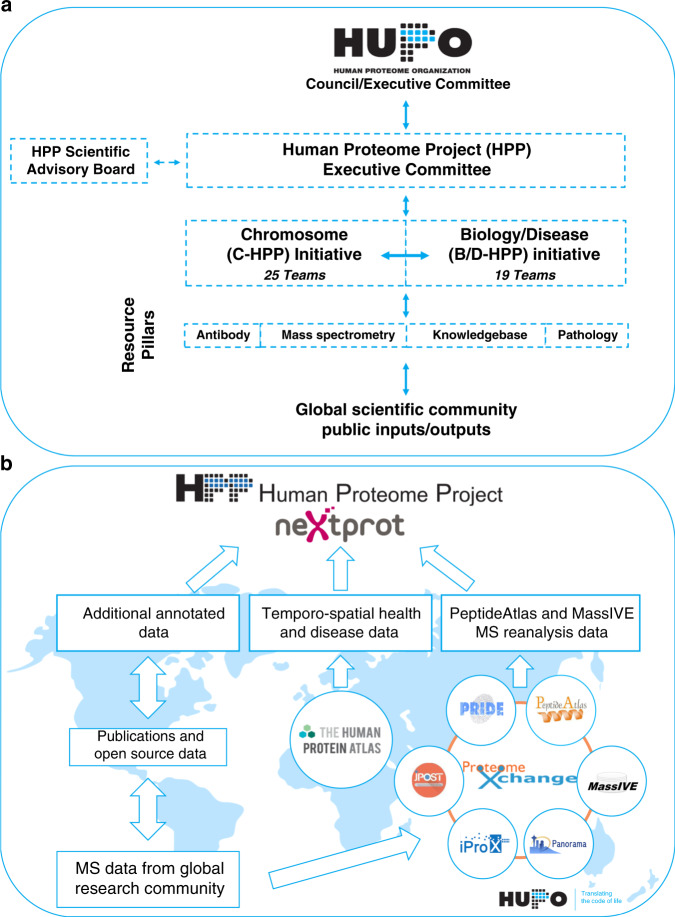
Structure of HUPO’s Human Proteome Project. **a** The HPP matrix formed by creating two major initiatives (C-HPP and B/D-HPP). The initiatives and their teams are underpinned by 4 Resource Pillars (AB, MS, KB and pathology). **b** The HPP KB pipeline demonstrates how MS, AB and other biological data are collected, processed, re-analysed and presented annually for FAIR (see below) use by the scientific community. MS datasets are deposited, tagged with a PXD identifier, and stored by PX repositories (PRIDE, PeptideAtlas, MassIVE, Panorama, iProX, JPOST). Data selection, extraction and re-analysis by PeptideAtlas and MassIVE results in processed data that is transmitted to neXtProt. Subsequently, neXtProt annotates and curates other biological data (like Sanger sequencing, protein : protein interaction and other structural/crystallographic data) that is aggregated, integrated and then disseminated to the community. The HUPO HPP KB uses reverse date versions (e.g., the latest 2020 neXtProt HPP reference release 17-01-2020).

The HPP grew from several archetypal HUPO projects (plasma, liver, brain, cardiovascular, kidney/urine proteomes). At present, the HPP comprises two strategic initiatives—chromosome-centric (C-HPP; 25 teams) and biology/disease-centric (B/D-HPP; 19 teams)—organized in a strategic matrix underpinned by four Resource Pillars: antibodies (AB), mass spectrometry (MS), KB and Pathology (Fig. 1a). Before explaining these elements in more detail, we will describe HPP’s criteria underlying the establishment of the high-stringency human proteome.

### Defining the human proteome at high-stringency

The HPP relies upon a coordinated system developed by neXtProt and UniProtKB, which attributes five levels of supporting evidence for protein existence (PE)^6^. Evidence at PE1 indicates clear experimental evidence for the existence of at least one proteoform, based on credible identification by MS, Edman sequencing, X-ray, nuclear magnetic resonance (NMR) structure of purified natural protein, reliable protein–protein interaction and/or antibody data. PE2 indicates evidence limited to the corresponding transcript (cDNA, reverse-transcriptase PCR, northern blotting). PE3 indicates the existence of orthologs in closely related species. PE4 refers to entries based on gene models without evidence at protein, transcript or homology levels. PE5 classifications indicate that coding evidence is doubtful and/or probably corresponds to an incorrect in silico translation of a non-coding element. As PE5 entries are largely non-coding, the HPP preferentially tracks only PE1,2,3,4 protein-coding entries. Proteins classified as PE2,3,4 are colloquially referred to as missing proteins^7,8^. Since 2013, neXtProt and the HPP (Fig. 1b) have issued annual tallies of PE1,2,3,4,5 status (www.nextprot.org/about/protein-existence), which have been reported in annual collaborative metrics manuscripts (ref. ^9^ and references therein).

The latest neXtProt HPP reference release (https://www.nextprot.org/about/statistics, data release: 17-01-2020) designates credible PE1 evidence for 90.4% of the human proteome (17,874 PE1s from the 19,773 PE1,2,3,4 protein entries excluding the dubious 577 PE5 entries). This leaves 1899 (9.6%) PE2,3,4 missing human proteome entries to be identified at high-stringency.

Here, the term high-stringency refers to rigorous HPP standards for post-acquisition processing and any protein inference made from raw MS peptide spectral data^10^. This term avoids confusion with the pre-existing term ‘high-accuracy’ frequently used in MS parlance, which relates to the generation of high (mass)-accuracy spectra from modern instruments.

The use of high-stringency influences the quality of all protein inferences derived from any raw MS data. The HPP routinely applies high-stringency protein inference analytics^10^, generated through the Trans-Proteomic Pipeline^11^. Claims for detection of new PE1s and/or detection of coding elements not previously in neXtProt, should meet minimum evidence thresholds. The current HPP guidelines^10^ require at least two uniquely mapping peptides (using neXtProt’s Peptide Uniqueness Checker Tool^12^), which are at least nine amino acid residues long. Peptides must be non-nested (i.e., one not fully contained within another) but may overlap partially so coverage exceeds >18 residues. The HPP also requires full declaration of false discovery rate (FDR) calculation procedures at peptide and protein levels, with a maximum allowable protein-level FDR of 1%. Within HPP missing protein publications, further validation is required through synthetic peptide spectra matching^13^. Going forward, the HPP also requests association of Universal Spectrum Identifiers with every MS spectrum^10^.

We emphasize that MS-based data from high-accuracy^14,15^ MS instruments combined with subsequent high-stringency protein inference analysis provide definitive best-practice confirmation of protein identification and abundance. To ensure high-quality analyses, increasingly stringent criteria have been applied^10,16^ and the latest HPP MS Guidelines v3.0^10^ are designed to make spectral data Findable, Accessible, Interoperable and Reusable (FAIR)^17^.

Many previous studies use high (mass)-accuracy instruments with subsequent protein inference identifications undertaken at lower default settings, such as accepting single peptides or those only seven amino acids in length and/or not conforming to more rigid neXtProt proteotypic analysis^18,19^. These analyses can result in spurious identification of many more false positives^20^, with lower-quality single non-proteotypic spectra data colloquially referred to as one-hit wonders, better explained by sequence variation in other highly observed proteins^21^.

### Chromosome-centric (C)-HPP

The C-HPP (https://www.hupo.org/C-HPP) aims to annotate all genome-encoded proteins^7,8^ in an unbiased and high-stringency manner. It explores proteins that have not previously been confidently observed by MS or other analytical methods^7,8,22^. International C-HPP teams are organized according to chromosomes (Chrs), namely Chrs 1–22, Chr X, Chr Y and mitochondrial (Mt) genome teams.

From 2017, the C-HPP expanded its mission to include functional characterization of the 1899 PE2,3,4 proteins that have not been confidently observed and 1254 PE1s that have no neXtProt curated function (uncharacterized PE1s or uPE1s), cumulatively referred to as the dark proteome^23,24^.

### Biology/disease-centric (B/D)-HPP

The B/D-HPP (https://www.hupo.org/B/D-HPP) measures and interprets human proteome data under a range of physiological and pathological conditions. It focuses on the following: (i) elucidating the hallmark protein drivers of biology/disease and (ii) promoting development of new proteomics analytical tools such as Ab-based approaches and targeted selected/multiple/parallel reaction monitoring (SRM/MRM/PRM) assays.

As an example, the initial HUPO liver proteome project grew into a B/D-HPP team focussing on liver expression profiles, PTMs, tissue expression, subcellular localization, interactions, physiology and pathologies^25^. The Chinese CN-HPP have characterized four liver cell types, emphasizing benefits of acquiring cell-type specific maps to understand underlying biology/pathology^26^. In addition, they mapped landscapes of early hepatocellular and lung carcinoma, generating cancer subtype alterations where proteomic signatures identified poor prognosis patients and/or those benefiting from targeted therapy^27,28^. In other studies, they analysed microdissected cell types with gross anatomical resolution using MS^26,29^, revealing circadian cycles and spatio-temporal proteome expression in the liver, brain, heart and stomach^30^, providing resources to better understand organ biochemistry, physiology and pathology.

Significant discoveries continue to be made from all B/D-HPP teams across personalized cancer immunotherapy and therapeutic modalities (e.g., lymphoma^31^, ovarian^32^, liver^27^ and lung^28^ cancers), with PTMs orchestrating many outcomes including response to therapy^33–35^.

B/D-HPP resources include the Human SRMAtlas^36^, a unique compendium of high-resolution spectra and multiplexed SRM/MRM/PRM assays developed from 166,174 synthetic proteotypic peptides. This assay library enables targeted identification and quantification of a theoretical maximum of 99.7% of the human proteome^36^, provided proteins are expressed spatiotemporally at concentrations amenable to MS detection. For example, SRMAtlas supported the C-HPP Chr X team’s confident identification of missing proteins^37^.

### HPP resource pillars

The B/D-HPP and C-HPP are supported by four HPP Resource Pillars that ensure effective data generation, integration and implementation, including the establishment of metrics and guidelines, enhancement of technology platforms, reagent development and optimal use of existing and emerging data streams (Fig. 1a).

The HPP MS Resource Pillar informs the community about MS technology/workflow advances, appropriate high-stringency standards and liaises with industry regarding instrument development, all leading to improved depth and accuracy of proteome identification, quantification and modification. These include methods like matrix-assisted laser desorption ionization time of flight (MALDI-TOF)-MS, electrospray-MS, bottom-up (shotgun) MS, data-dependent acquisition MS, data-independent acquisition (DIA) MS, targeted SRM/MRM/PRM, top-down MS, cross-linking MS, PTM analysis, N- and C-termini measurement, MS data computational analysis and interactomics.

The MS Resource Pillar previously undertook a SWATH/DIA-MS reproducibility study^38^ and are currently coordinating a phosphopeptide challenge involving >20 participating labs with partners SynPeptide Shanghai and Resyn Biosciences South Africa, who have provided a human phosphopeptide standard set with unphosphorylated counterparts, as peptide mixtures spiked into a yeast tryptic digest background. This will result in a better understanding of phosphopeptide enrichment, MS data analytics and informatics tools.

The HPP Ab Resource Pillar, ostensibly led by the Human Protein Atlas (HPA; www.proteinatlas.org), was initiated in 2003 and uses Ab-based strategies to analyse spatio-temporal aspects of the proteome^39^. Linking the identification of proteins with ‘real-time’ localization at tissue, cell and subcellular levels supports a more comprehensive understanding of biology, health and disease. This requires information at resolution not currently available by MS (see single-cell section below). Approaches for spatio-temporal proteomics include single-cell in situ MS, fractionated cell lysates, proximity labelling or imaging-based proteomics^40,41^. Imaging-based proteomics has a clear advantage, namely analysing proteins in their native location at single-cell resolution. To this end, the HPA has developed industrial scale epitope-directed Abs for community use.

HPA also integrates multi-omics data. It contains extensive transcriptome data and neXtProt PE assignments, and contributes to the open-access catalogue Antibodypedia, containing >4 M Abs (www.antibodypedia.org)^42^ against >19,000 targets that assist the community to select application-appropriate Abs.

At HPP launch, the HPA had detected >50% of the protein-coding genome^43^. Currently, ~87% of the proteome is targeted by >1 HPA Ab, detected though an encyclopaedia of >10 M annotated high-resolution digital images, partitioned into a number of sub-atlases that are interconnected^44–48^. These currently comprise the; Tissue Atlas (protein distribution across all major tissues), Cell Atlas (subcellular localization and heterogeneity in single cells), Pathology Atlas (correlations between gene expression and patient survival in major human cancer types), Blood Atlas (protein profiles across major immune cells and blood levels), Brain Atlas (protein distribution in the brain), and the Metabolic Atlas (various tissue metabolic enzymes localizations). Over the decade, HPA’s open-access database^44–48^ has become one of the world’s most visited biological resources (>3.6 M visits in toto annually).

The HPA also plays an emerging role in establishing guidelines around the appropriate use of Abs and ensuring immunoassay validation^49–51^. It recently spent considerable effort validating the selectivity of their Abs, including championing efforts of the International Working Group for Ab Validation proposing many new approaches implemented across >10,000 Abs^42^. In addition, HPA’s massive collection of images has supported a multitude of publications and become a citizen science resource for developing AI classification learning models^52,53^. The HPA is sustained through community contribution to ELIXIR^54^, that allows scientists from academia and industry to explore spatio-temporal aspects of the human proteome^55–57^.

Since 2018, the HPP Pathology Resource Pillar has coordinated identification of areas of unmet clinical need, develops fit-for-purpose clinical assay guidelines/standards, promotes best-practice awareness, coordinates access to quality clinical samples/metadata and liaises with pathology organizations, diagnostic companies and regulatory agencies to promote professional application of proteomics in pathology.

The HPP KB Resource Pillar captures, collects, collates, analyses and re-distributes all human proteome data. As cohesive knowledge transfer plays such a crucial role in big data science, including the HPP, the KB pillar’s activities are addressed in the expanded section that follows.

## The human proteome in the neXtProt HPP reference KB

### Assembly and curation of neXtProt

Prior to the HPP, HUPO established a Protein Standards Initiative (PSI; www.hupo.org/Proteomics-Standards-Initiative), emphasizing from the outset their priority for defining high-quality community standards, minimal requirements for experimental information^58^ and high-stringency data metrics. PSI continues to work cooperatively with the HPP KB to inform HPP initiatives, pillars and teams.

In 2013, neXtProt^59,60^ was officially designated as the HPP reference KB^61^. Annually, a neXtProt release is designated as the ‘HPP release’ and this serves as the basis for subsequent HPP high-stringency analyses, planning and reporting progress^10^. It receives and curates data from UniProtKB/SwissProt^62^, adding MS evidence from PeptideAtlas^63^ and since 2019 from MassIVE^64^. neXtProt also curates Ab-based, genome, transcriptome and other biological data to create an assembled snapshot of the human proteome^6,65^.

PeptideAtlas (http://www.peptideatlas.org) uses sequence search engines Comet^66^, X!Tandem^67^ and SpectraST^68^ to reprocess publicly uploaded MS/MS data deposited through ProteomeXchange (PX). Data are aggregated using rigorous criteria including peptide spectral matching with FDR ~ 0.0009% in the latest PeptideAtlas build to achieve ≤1% FDR at the protein level. MassIVE searches public datasets using the MS-GF+ search engine^69^, also with strict criteria^70^ to enforce global <1% FDR at the protein level for single-peptide identifications and stricter <0.01% FDR for proteins identified by >2 peptides. PeptideAtlas and MassIVE peptide lists are integrated into current neXtProt builds. neXtProt then cross-references all peptides to protein entries and validates PE levels, requiring at least two MS-identified uniquely mapping 9-mer non-nested peptides coming from either PeptideAtlas or MassIVE.

neXtProt builds on UniProtKB/SwissProt PE1 entries that include MS, partial or complete Edman sequencing, X-ray or NMR structure, reliable protein–protein interaction data or Ab detection by considering additional PeptideAtlas/MassIVE data that meets minimum peptide uniqueness, number, length, nestedness and other requirements to upgrade entries to PE1. Noticeably, neXtProt is becoming more reliant on MS than non-MS data (e.g., 1860 non-MS data PE1s in 2016 down to 950 in 2020).

To ensure only high-quality Ab data are used, in 2018 neXtProt/HPP Ab pillar revised criteria to upgrade entries to PE1, including specificity and other rigorous criteria suggested by the International Working Group for Ab Validation^50^. As an example, neXtProt recently analysed 41 Ab-based publications and upgraded three PE2,3,4 entries to PE1. Discussions regarding data provenance delivered by non-MS data for PE1 assignment actively continues in the HPP^71^.

### Expansion of the high-stringency proteome

The baseline number of protein-coding genes in the reference proteome given by neXtProt is managed by UniProtKB/SwissProt. Every protein-coding gene is assigned a protein entry (inclusive of all proteoforms), with chromosome location and other data organized under these entries. The number of protein-coding genes originally estimated by the HGP dropped from >100,000^72^ to a relatively stable ~19,700 at HGP draft release, a number that is close to the current neXtProt’s 2020 release of 19,773 protein-coding genes (Supplementary Fig. 1)^60^.

**Fig. 2:**
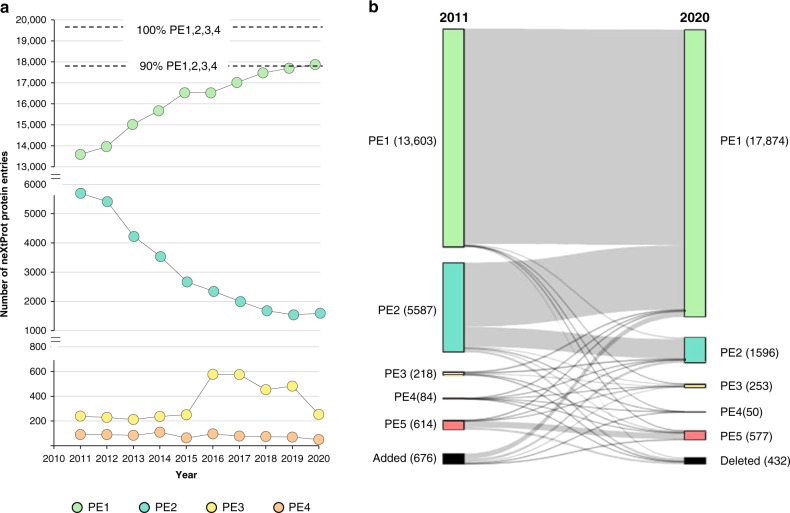
Completing >90% of the high-stringency human proteome. **a** Annual neXtProt HPP evidence of protein existence (PE1,2,3,4,5) metrics from 2010 to 2020. This data demonstrates a strong and progressive increase in PE1 identifications across the decade (13,588 in 2011 to 17,874 in 2020), correlative equivalent decrease in PE2 (5,696 to 1596), a post-2015 rise in PE3 coincident with revised guideline implementation (239 to 253) and decrease in PE4 identifications (90 down to 50). PMS Pantone colours employed match in the figure match for all past annual neXtProt HPP KB reference PE1,2,3,4,5 data release colours, namely PE1: light green, PE2: teal, PE3: yellow, PE4: orange, and PE5: red). **b** Decadal Sankey diagram of changes in PE1,2,3,4,5 status of neXtProt entries between 2011 and 2020, where arrow widths are proportional to the number of decadal PE entries that change category. This Sankey diagram displays fluidity in PE status of neXtProt entries. PMS Pantone colours match those used for all past annual neXtProt HPP KB reference PE1,2,3,4,5 data releases https://www.nextprot.org/about/protein-existence (i.e., PE1: light green, PE2: teal, PE3: yellow, PE4: orange and PE5: cerise). All neXtProt protein entries that were deleted or newly introduced during the decade are represented in black, noting that 432 neXtProt entries were deleted and 676 introduced. Sankey analysis demonstrates that 2011 PE2 entries were the most significant (but not exclusively) the source for the majority of additional 2020 PE1s. Year-by-year transition data can be found in metrics publications associated with annual (2013–2019) HPP special issues^9^ and refs therein, guided by high-stringency HPP Guidelines^10^.

Confident detection of the human proteome has consistently risen from 69.8% neXtProt PE1 entries in 2011 to 90.4% in 2020 (Fig. 2a). However, progress has recently slowed (Fig. 2a), suggesting that it may be difficult to confidently identify all 1899 missing proteins. Parenthetically, the HGP celebrated a provisional 90% completion ten years after its launch^5^ and annotation of the human genome still remains incomplete or uncertain, especially in regions of repeat sequences and Z-DNA inserts.

In each of the seven annual HPP metrics papers published to date (^9^ and refs therein), PE1,2,3,4,5 entries have been added, deleted, renamed, merged and/or de-merged, indicating ongoing fine-tuning of the HPP reference proteome. The Sankey flow diagram^73^ (Fig. 2b) illustrates that significant fluidity has occurred across all PE classifications since 2011. The largest shifts occur with increases in PE1s (13,603 up to 17,874), followed by decreases in PE2s (5,587 down to 1,596), despite increasingly stringent HPP MS data guidelines adopted in 2015 (v2.1) and 2019 (v3.0). Linear regression of PE1 increases against PE2 decreases results in a strong (R^2^ = 97%) inverse correlation, suggesting new PE1s come from PE2s where mRNA expression has been previously observed. Extrapolation of this PE1 discovery curve suggests that 95% PE1s may be reached sometime between 2024 and 2027. Minor conversions occurred between other PE categories, with downgrading of PE1 or PE2s and upgrading of PE4s, and even a few PE5s, to PE1s. Although a plethora of studies show low linear correlations (40–60%) between mRNA level and protein abundance^74,75^, our binary data (Fig. 2) supports the contention that once a neXtProt entry has mRNA expression verified, those PE2s are amenable to upgrade to PE1, whereas PE3s, PE4s and particularly PE5s are more resistant to PE1 upgrade.

### Missing protein analyses

**Fig. 3:**
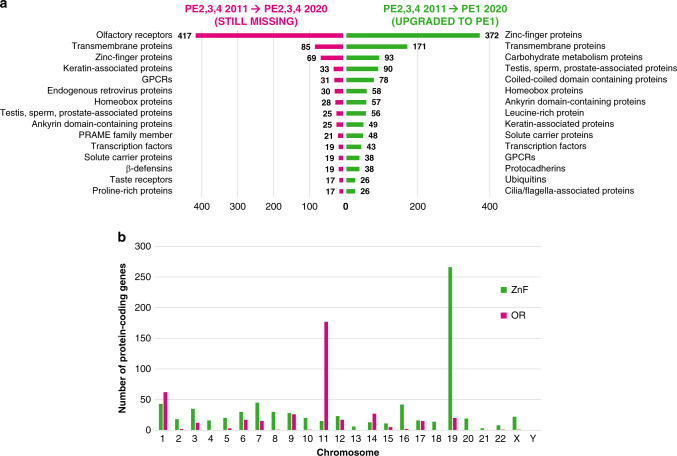
HPP decadal impact. **a** The top 15 neXtProt protein descriptor groups/families with the highest number of 2020 PE2,3,4 missing protein members (i.e., lacking high-stringency PE1 evidence of protein existence data; magenta, left) and the top 15 protein descriptor groups that have been upgraded to PE1 since 2011 (green, right). The data illustrates that the OR family has the highest number of 2020 missing PE2,3,4 proteins (magenta bars) and the Zn finger protein family has the highest number of discovered PE2,3,4 entries upgraded to PE1 since 2011 **b** Human chromosomal distribution of the OR and Zn finger families neXtProt protein descriptor groups/families. This example data clearly illustrates that the positioning of multiple ORs (magenta vertical bars) or Zn finger protein-coding genes (green vertical bars) on certain chromsomes explains why Chr 11 appears more resistant and Chr 19 more susceptible to PE1 discovery over the decade.

neXtProt protein descriptors and associated data can be used to analyse protein groups/families according to their Chr location or PE classification. Missing protein (PE2,3,4) analysis indicates that some groups/families have been upgraded to PE1 more successfully than others (Fig. 3a). For example, between 2011 and 2020, 372 zinc (Zn) finger proteins, 171 transmembrane proteins, 93 carbohydrate metabolism proteins, 90 testes-, sperm-, prostate-associated proteins, 78 coiled-coil domain-containing proteins and 58 homeobox proteins have been upgraded from PE2,3,4 to PE1. These represent the six most prominent protein groups upgraded to PE1 over the decade (Fig. 3a, green). In contrast, two G protein-coupled receptor (GPCR) chemosensory families prove particularly resistant to PE1 upgrade: many olfactory receptors (ORs) are still missing in 2020^76^ (417 of the 2011 PE2,3,4 entries, including putative and uncharacterized ones) and 17 of 20 taste receptors remain PE2,3,4 s (Fig. 3a, magenta). In addition, some groups with a large number of PE1-upgraded protein entries still contain many PE2,3,4 entries (e.g., 85 non-GPCR transmembrane, 69 Zn finger and 33 keratin-associated proteins remain PE2,3,4 s (Fig. 3a).

When Chr distribution of the most resistant (ORs) or most discovered (Zn-finger proteins) groups were plotted (Fig. 3b), difficult-to-find ORs mapped mostly to Chr 11 (~55%) and Zn-finger proteins mapped mostly to Chr 19 (~55%). Our data demonstrate that 46% of all Chr 19 proteins elevated to PE1 were Zn-finger family members, illustrating this protein family has been highly amenable to confident MS detection over the decade. Therefore, it was not surprising that most Zn-finger protein members were coded on Chr 19 (i.e., 255/698 total Zn-finger genes), which has the highest Chr PE1 reclassification rate over the decade (Fig. 3b).

**Fig. 4:**
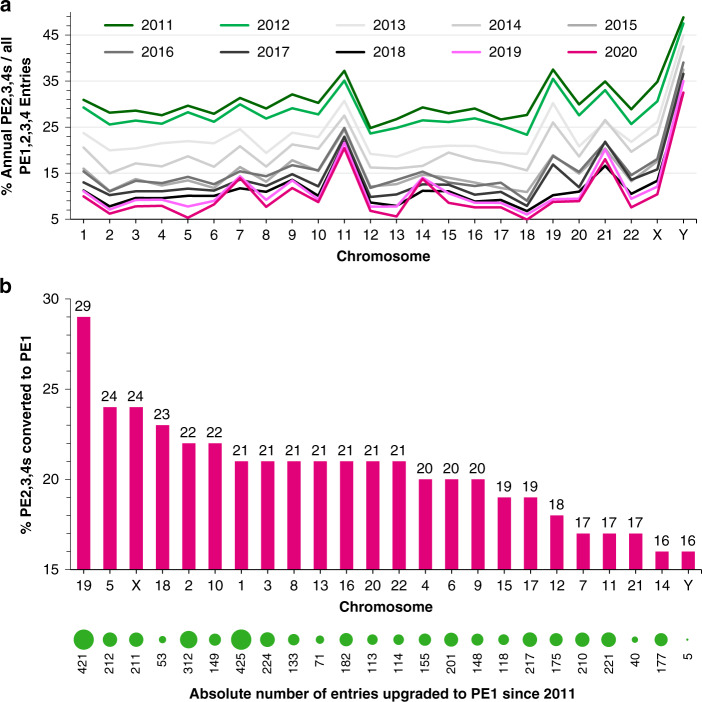
Progress in reducing the fraction of missing proteins for all human chromosomes. **a** The percentage of missing proteins (PE2,3,4) relative to all protein-coding genes (PE1,2,3,4) plotted annually according to human Chrs 1–22, X and Y location from the first neXtProt release (23-08-2011) to the latest HPP reference release (17-01-2020). **b** The relative percentage (magenta bars) and absolute number (green dots) of all neXtProt PE2,3,4 missing protein entries specifically upgraded to PE1 since 2011 across Chrs 1–22, X and Y.

These data suggest that gene family duplication on particular Chrs explains why some families are resistant or sensitive to PE1 reclassification. In agreement, PE1 discovery has occurred productively, but not uniformly, across all chromosomes (Fig. 4). Missing protein decadal upgrade statistics range from 16% for Chr Y up to 29% for Chr 1, with raw data representing 425 protein entries for Chr 1 down to only 5 for Chr Y. A higher percentage of Chr 19 proteins (29%) ascended to PE1 compared to those on Chrs 11, 14, 21 or Y (<17%). The HPA chromosome viewer (www.proteinatlas.org/humanproteome/proteinevidence) illustrates many recently-evolved protein family members are present on Chr Y, many ORs on Chr 11 and many keratin-associated proteins on Chr 21. Proteins on these three Chrs have proven relatively resistant to PE1 reassignment. Notably, there was just a single Mt missing protein in 2011 and over the decade all Mt protein-coding genes have now been identified.

PE1 upgrade success by the 25 C-HPP teams could be pre-determined by the presence of resistant or more easily-identified missing protein families on particular Chrs. However, the current HPP KB processing pipeline utilizing PeptideAtlas, MassIVE and neXtProt (Fig. 1b) makes it impossible to isolate decadal PE1 contributions from any particular C-HPP team as opposed to the overall community. Although the HPP explores capturing full data provenance (i.e., from quantification/identification back to original data source) for FAIR data practices^17^, we can only historically estimate PE1s emanating from community deposition.

In silico analysis reveals only 22 human proteins cannot produce the characteristic >2 high-stringency proteotypic peptides of the required length after tryptic digestion^36^. However, many missing proteins may be present at levels below detection limits or in under-studied cell types/tissues, expressed under particular conditions (e.g., stress/infection) or only found in developmental stages (e.g., embryo/fetus)^77^. Equally, difficult-to-solubilize, hydrophobic multi-transmembrane domain membrane proteins may only generate short tryptic peptides that do not meet high-stringency guidelines or are indistinguishable from other family member sequences^76^. Furthermore, transmembrane protein regions cut at single sites are unlikely to release embedded hydrophobic membrane-anchored protein strands^76^.

We anticipate that finding the 9.6% remaining PE2,3,4 missing proteins will require exceptional future effort, including careful sampling of rare cells/tissues^78^ combined with better sample fractionation and improved detection limits. Low abundance proteins might be enriched using Abs prior to MS. To this end, the HPA has developed Abs against proteotypic sequences in many missing proteins^79^. Several other labs are working on improved protocols for insoluble keratin-associated cross-linked missing proteins, non-tryptic or chemical digestion strategies to increase proteotypic peptide productivity^78,80^, higher efficiency search engines^81^, and compendia of missing protein biological evidence (e.g., MissingProteinPedia)^71^. Future HPP projects anticipate a shift to detection of biologically functional proteoforms^82^, noting their numbers are far larger and more difficult to measure^83^ because of heterogeneous nuclear RNA splicing, many PTMs and detection of peptides with single amino acid variants (SAAVs). Considerable PTM and splicing isoform data are already available through neXtProt, including 190,938 PTM sites and 9,193,365 SAAVs^84^.

## Community impact on the human proteome

One barometer of community engagement in the HPP is the magnitude of investigator-submitted raw MS data that have been re-analysed. Many journals require raw data submission and HPP action was a significant factor in journals adopting requirements aligned to HPP data deposition guidelines. Raw data deposition occurs through PX, which registers and standardizes capture/dissemination of public MS data from partner repositories, including founders PRIDE^62^ and PeptideAtlas and recent members MassIVE^64^, jPOST^85^, iProX^86^ and Panorama Public^87^. As of 2020, a total of 4634 human MS datasets have been received. Each PX dataset is branded with a unique PXD identifier with depositors, publications and voluntary metadata noted^88,89^. Illustrating the magnitude of this community data, ~470 TBs of data have come from 5658 human datasets (~47% of 1 petabyte PRIDE volume), with only 358 (6.3%) of these specifically tagged by depositors as from the HPP. The HPP encourages raw human MS data/metadata submission (including association to HPP) through PX, and that journals request that PXD identifiers be published in accordance with FAIR principles^17^ as discussed above.

To provide an additional measure of global scientific impact, HUPO commissioned the website construction of the Human Proteome Reference Library (HPRL; https://hupo.org/HPP-HPRL/), where all HPP-associated PubMed searches are hyperlinked and can be accessed and re-run routinely by the community. These hyperlinked searches automatically produce the latest PubMed outputs in a manner where all PubMed filtering, ranking and timeline tools can be applied subsequently by a user as required. As an example of the fascinating data unearthed, a ‘human proteome project’ search showed that the structural biologists Montelione and Anderson first suggested the possibility of building a HPP in their 1999 Nature Structural Biology publication^90^—well before HUPO or the HPP began.

**Fig. 5:**
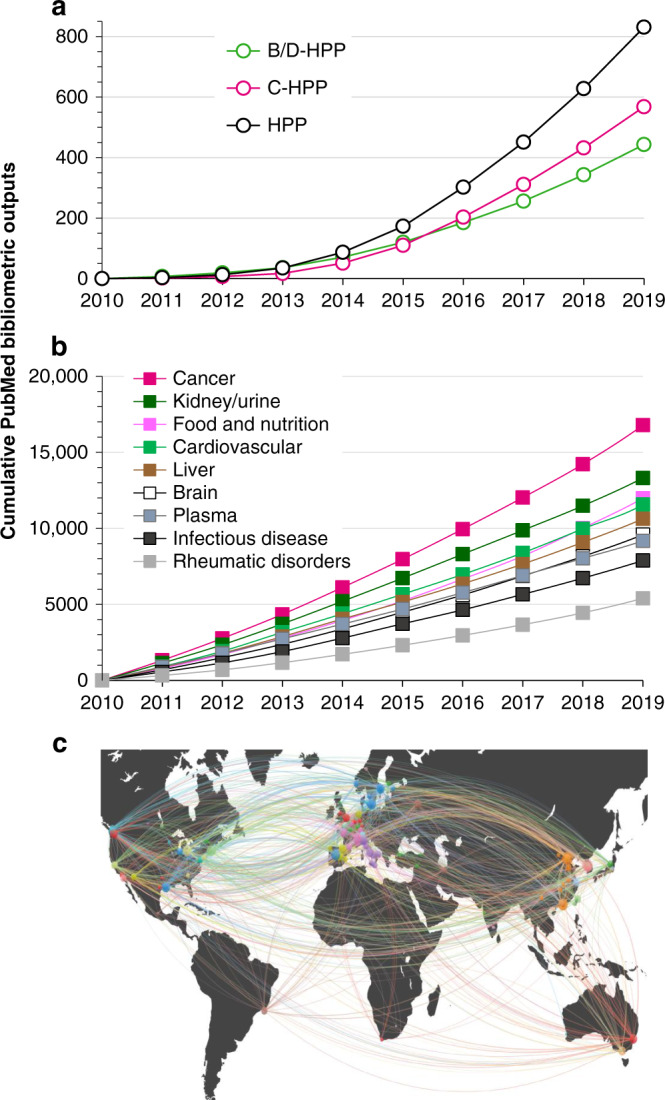
Assembly of the Human Proteome Reference Library (HPRL). Data show cumulative PubMed search references emanating since HPP launch in 2010 up until 2019. **a** PubMed search for the terms HPP, C-HPP and B/D-HPP (including unabbreviated version). **b** PubMed community-at-large bibliometric impacts that parallel the research disciplines (e.g., ‘human’ AND ‘cancer proteomics’) addressed and undertaken by key B/D-HPP teams. All B/D-HPP PubMed bibliometric searches are listed as full searches and as hyperlinked current PubMed searches on the HUPO website at https://hupo.org/HPP-HPRL/. All NCBI PubMed filters and tools are fully accessible to users and searches can be selected and modified in a user-friendly manner, allowing decadal (from 2010 to 2019) and other bibliometric analyses to be undertaken routinely. **c** VOSviewer HPP collaborations analysis. All co-author geographical affiliations for PubMed publications emanating from Fig. 5a were transposed onto a world map.

The HPRL can also be used to measure impact (Fig. 5), where PubMed identifiers (PMIDs) from 2010 to 2019 references can be captured using web APIs or software, e.g., NCBI E-utilities like https://www.ncbi.nlm.nih.gov/books/NBK25501 or the easyPubMed R interface (www.rdrr.io/cran/easyPubMed) that enable extraction and aggregation of PubMed bibliometric records. Paralleling the low percentage of PX datasets tagged as emanating from the HPP, HPRL data show only ~1000 PubMed title and abstract searches that specifically and cumulatively mention either the terms HPP, C-HPP and/or B/D-HPP (or their unabbreviated counterparts) out of a total of more than 50,000 ‘human AND proteomics’ title/abstract search outcomes over the decade. In contrast to this and more reassuringly, HPRL searches of the community’s biology/ or disease studies covered by nine of the B/D-HPP teams generated >5000 publications each since 2010 (Fig. 5b), with ‘cancer proteomics’ topping rankings at around 20,000 publications. Although this may seem surprising, a PubMed search of ‘human genome project’ produced a similarly modest number of ~1900 hits during the HGP’s first decade. To visualize outputs from various HPP teams and the proteomics community in general, we have employed VOSviewer^91^ to construct and visualize bibliometric networks from collective PubMed searches (example shown in Fig. 5c). This particular analysis revealed a remarkable number of highly-interconnected relationships that have evolved between HPP teams, pillars and initiatives—providing evidence for the impressive level of established international collaboration developed over the decade.

## Translating proteomics to precision medicine

A key aspect of biomedical research lies in translating discovery into clinical use. Protein assays remain a cornerstone of diagnostics. Although individual proteins can be measured diagnostically with high precision (i.e., sensitivity and specificity), some assays suffer low specificity due to cross-reactivity with interfering substances including autoantibodies (e.g., thyroglobulin immunoassays). Modern SRM/MRM/PRM assays allow multiple proteins to be measured simultaneously, accurately, sensitively and with high specificity. In addition, the use of liquid chromatography MS with immunocapture assays has been reported to eliminate interferences^92^. Moreover, as most diseases are heterogeneous and multigenic, it is likely that multiplexed proteomic or multi-omics panels will achieve higher accuracy (e.g., optimized biomarkers for ovarian malignancy with adnexal masses^93^). The HPP assists in the development of proteomic educational programmes with pathology societies to train the pathology community on the potential impact of proteomic technologies.

Below, in recognition of the impact human proteomics can and is having in precision medicine, we highlight examples demonstrating the role of the HPP and proteomics in tackling contemporary medical grand challenges.

### Cancer precision medicine

As mentioned above, PubMed extracts ~20,000 published human cancer proteome studies since 2011 (Fig. 5b). Although genomics can routinely determine high-risk, predisposition and aspects related to tumour burden and recurrence, effective targeted cancer treatment is still not available for all cancers. For example, systematic genome-wide studies like the Pan-Cancer Analysis integrated analyses of >2600 whole genomes from 38 tumour types with matching normal tissues, uncovered many cancer-associated genes^94^, chromosome rearrangements, some unknown drivers but few new therapeutic targets. This is mainly because mutations do not automatically cause predicted changes in the proteome, making it difficult to establish which changes are crucial biochemical drivers from those that are not.

Integrating genomic and proteomic data (i.e., proteogenomics) has the potential to provide insights into causes and mechanisms underlying diseases, including the hallmarks of cancer biology^95^. This can facilitate the implementation of effective therapeutic intervention. The value of a proteogenomic analysis of functional consequences of cancer somatic mutations has assisted in narrowing down candidate driver genes within large deletions and amplified regions^96^. Reviewing the underlying causes of breast cancer also demonstrates that coupling genomic/transcriptomic data with proteomic/phosphoproteomic analysis was more insightful than any individual approach. Of note, melanoma tumour genomic BRAF driver mutations match corresponding protein sequences^97^, illustrating that proteomic landscapes add value to genomic data, when considered with patient tumour histopathology and clinical metadata^98^.

Proteomics substantially benefits a comprehensive understanding of precision medicine. To illustrate this, NCI’s Clinical Proteomic Tumor Analysis Consortium (CPTAC; proteomics.cancer.gov) in collaboration with the B/D-HPP cancer team, established guidelines, data sharing, and standards in analytical and computational workflows to ensure rigour in designing and performing research^99–103^. CPTAC applied these standards and workflows to tumours previously genomically characterized by The Cancer Genome Atlas. In doing so, CPTAC pioneered the integration of proteomics with genomics (i.e., proteogenomics) to produce a more unified and comprehensive understanding of cancer biology and implemented its transition into cancer clinical research studies^96,104,105^. Largely due to these efforts, NCI hosts open-access repositories of unified proteogenomics datasets, assays and reagents, including the Proteomic Data Commons (pdc.cancer.gov), a fit-for-purpose targeted assay site (assays.cancer.gov)^102,106^ and an Ab portal (antibodies.cancer.gov)^107^. These activities empowered the recent creation of the International Proteogenome Consortium (ICPC; icpc.cancer.gov)^108^. Collectively, CPTAC and ICPC collaborators have comprehensively characterized 13 cancer types at the proteogenomics level, with all datasets publicly accessible^109–117^.

### Cardiovascular diseases

Cardiovascular disease (CVD) research is challenged by daunting structural heterogeneity and molecular complexity. For example, cardiac circuitry function/dysfunction cannot be reduced to differentially expressed single genes. On the other hand, proteogenomics allows assessment of interactions, pathways and networks and informs diagnosis and therapy of multifactorial CVDs. Over the decade, CVD proteomics has broadened from identifying single canonical proteins to mapping proteoforms derived from combinations of alternative splicing, cleavage, and PTMs^33,83^. Now, identification of proteoforms (e.g., genetic variations, alternatively spliced products, phosphorylation^118^, glycosylation^119^, oxidative^120^ and other PTMs^121^) allow CVD sub-classification. In parallel, the heart is uniquely sensitive to alternative splicing (frequently altered in congenital heart diseases), explaining the B/D-HPP’s interest in developing assays for splice isoform-specific changes in cardiomyocyte development and maturation^122,123^.

Many technological developments have been prominent, including phospho-PTM analysis to identify PDE5A targets in heart failure therapy^118^ and proximity labelling to assess protein–protein interactions involved in β-adrenergic signalling of contractility in cardiac fight-or-flight responses^124^ and evaluation of regenerative stem cell therapy efficacy in post-infarct hearts^125^. Proteomic studies have also addressed the kilometres of vascular beds and extracellular matrix responsible for transporting blood, giving valuable insights into the molecular anatomy of aneurysms and atherosclerosis^126,127^. Targeted MS methods have been developed, again especially where interferences obfuscate immunoassays^128^ or where additional biological context is required^129^. Likewise, volumetric absorptive micro-sampling VAMS blood collection devices (e.g., Mitra devices or dried blood spots) allow patients to mail samples from home to analytical labs to undertake SRM/MRM/PRM assays quantifying CVD risk-associated apolipoproteins^130^ or other markers^131^. In summary, consumer-based CVD proteomics-based precision medicine testing services are now coming of age.

### Microorganism detection

Proteomics and the HPP have made fundamental contributions to understanding pathogenic infection, providing diagnostics and developing therapies^132^. The B/D-HPP Infectious Diseases team promotes international proteomics collaborations investigating viral, bacterial, fungal and parasitic diseases.

MALDI–time-of-flight (TOF)–MS, once considered revolutionary, is now established as a routine tool in clinical microbiology^133^. Classical phenotypic tests identify unknown and potentially pathogenic microorganisms, but may require incubation for several days, with misidentification resulting in adverse treatment consequences. MALDI–TOF–MS provides significantly shortened analyses (now minutes) with improved accuracy on single colony or bacterial pellets for difficult-to-detect microorganisms, using automated spectra acquisition and extensive reference spectra databases^134^. Minor spectral differences enable typing below species levels^135^, allowing subspecies identification through epidemiological analyses. Bacteria and yeasts (most clinical identifications), mycobacteria^136^ and moulds^137^ can now be identified accurately and rapidly. Further MS clinical diagnostic applications are being investigated (e.g., antibiotic resistance (ART) and susceptibility testing (AST) based on hydrolytic β-lactamase activity^138^), with kits under development commercially through STAR-Carba, STAR-Cepha and Bruker Daltonics.

### SARS-CoV-2 virology

The recent severe acute respiratory syndrome coronavirus 2 (SARS-CoV-2) outbreak that causes COVID-19 disease represents a major threat to human health and our economies^139–141^. The pandemic underscores our need to understand virus pathobiology, identify host-pathogen interactions that support replication, find biomarkers correlative with clinical outcome and expand surveillance.

Many omics studies followed the 2003 SARS-CoV-1 and related MERS and IBV coronavirus outbreaks^142–146^. The cell surface receptor for the CoV-1 and CoV-2 surface spike protein has been identified by affinity-MS to be angiotensin-converting enzyme 2 (ACE2)^147^, which in a recent large-scale study based on antibody-based proteomics was shown to be mainly localized to the digestive system, kidney, heart, testis, placenta, eye and upper respiratory epithelia^148^. Virus binding leads to proteolysis by the transmembrane serine protease TMPRSS2 expressed in airway epithelia^149^, thus a clinically approved TMPRSS2 inhibitor (camostat mesylate) is being investigated to block infections^150,151^. Furthermore, proteomics has characterized the infectious CoV-1 viral particle^143^, temporal changes in host cells during infection^142^ and virus-induced endoplasmic reticulum membrane remodelling into double-membrane vesicles^152,153^ that house viral replication compartments^146^. Proximity labelling revealed >500 host and 14 viral protein associations with the viral replicase NPS2^146^, highlighting vesicular trafficking, autophagy and splicing proteins in coronavirus replication, which if shown to also be the case for CoV-2, indicate potential drug targets.

Building on this knowledge of coronavirus infection, recent proteomics studies have focused on SARS-CoV-2, uncovering additional potential therapeutic targets^154,155^. MS and array-based proteomics serology has screened for potential biomarkers and Abs against infection^156,157^. Clinical isolate infection models have been developed using Caco-2 cells^154^ with temporal proteome changes identified during infection using multiplexed MS by combining metabolic labelling with tandem mass tagging methods. Consistently, host vesicular trafficking, translation, RNA splicing, nucleotide synthesis and glycolysis pathway proteins were upregulated following infection^143,144^ and targeting these processes with inhibitors revealed potential therapeutic targets^158,159^. Additionally, affinity-MS interactome studies examined 26 of 29 total SARS-CoV-2 proteins expressed within HEK293T human cells^155^, suggesting 69 existing drugs merit further investigation. Moreover, a recent phosphoproteome analysis pointed to the regulation of viral proteins through PTMs^160^.

The SARS-CoV-2 pandemic highlights the need for applying proteomic approaches to the development of serologic testing and preclinical and computational model systems to evaluate patient responses to infection^156^. Serological biomarkers of asymptomatic/symptomatic infection, disease severity, risk of re-infection and/or vaccine efficacy are being characterized^157,161^. In addition to this accumulating omics knowledge, many aspects of SARS-CoV-2 pathobiology await further exploration including development of additional methods for clinical virus detection, identification of infection stage, and an in-depth understanding of functional spatio-temporal virus–host protein interactions and organelle remodelling^162–164^. For example, recent studies that utilize targeted MS for SARS-CoV-2 protein detection and proteomic characterization of serological immune responses^161,165–167^ from patient samples may bolster PCR screening for the assessment of disease severity^168^. Other proteomics approaches can also be deployed to further expand the understanding of SARS-CoV-2 biology. Among these is the application of TAILS N-terminomics that promises to identify many SARS-CoV-2 protease substrates and those cellular pathways inactivated by viral proteolysis, as reported for other viruses^169^.

In summary, proteomics plays increasingly important roles in understanding viral outbreak biology, accurate diagnosis and effective treatment and is positioned to continue to co-ordinate and drive international collaborations towards these goals.

## Conclusions and future directions

Western and Eastern cultures urge us to know thyself and thy enemy. These axioms resonate with precision medicine where future benefits arise from a detailed omics understanding of the hallmarks of health and disease. Here, we reviewed the construction of a community-endorsed, high-stringency blueprint of the human proteome. The decadal neXtProt HPP PE metrics shown here demonstrate the community’s progressive success in PE1 identification from 13,588 in 2011 to 17,874 PE1s in 2020, marking the completion of >90% of the human proteome parts list (see strategic objective 1 above). We also present specific examples demonstrating proteomics will be an integrated component (with genomics and other omics) in future biomedical science discovery and precision medicine.

HUPO recommits to its original HPP strategic aims as well as the FAIR data principles^17^, while anticipating the following future priorities:

1. Unearth credible proteomics data for the majority of current PE2 proteins: Since most PE1 identifications come from former PE2s, our future strategy is to find credible data for 95–99% of current PE2s, allowing reclassification of these to PE1. PE3 also remain promising, as homologous proteins are detected in related species.
2. Unravel currently unknown proteome functionalities: Fill functional annotation gaps for all protein-coding elements, with a priority on credibly identified proteins^170^ and develop, expand and apply function prediction tools^24,171,172^.
3. Expand the HPP KB: Maintain a sustainable knowledge-transfer HPP KB infrastructure with funding that captures/displays high-stringency partner omics data streams and publication data (HPRL) to researchers and the public in an accessible and compelling manner.
4. Develop community-approved multi-omics technology guidelines: Explore DIA-MS, Ab and aptamer-based multiplexed assays, top-down MS and other not yet invented multi-omics technologies.
5. Champion collaborative multi-omics health/disease approaches: In addition to extensions in HPP KB partnerships, the HPP will collaborate with international and regional initiatives (including Human Variome, Human Cell Atlas, hPOP/iPOP, MoTrPAC, HuBMAP, Cancer Moonshot, HTAN, EDRN, CPTAC, ICPC and upcoming international initiatives) around multi-omics approaches to human disease processes, biomarker discovery and therapeutic development.
6. Apply and improve single-cell proteomic technology: Develop technologies that allow detection and quantification of proteomes in single cells to further understand cellular/tissue heterogeneity, differentiation, diseases and the intrinsic biological noise in health and disease^173,174^. Many single-cell proteomics advances will be explored to analyse cellular heterogeneity^175–178^. Sensitivity increases (analogous to PCR) and trade-offs maximizing coverage per cell with throughput/accuracy will be studied^179^.
7. Champion dual Ab-capture with MS identification: Enhance the accuracy of Ab-based epitope/antigen detection by confirmation using high-accuracy, high-stringency MS identifications across real-life spatio-temporal biological settings. Collectively, there is recognition within the HPP that future stringent co-registration of MS with Ab data are required to achieve the ultimate spatio-temporal human proteome expression atlas^180^.
8. Exploit massively parallel MS^174^ to increase throughput: For rapid, sensitive and higher-throughput MS analysis.
9. Capitalize on human disease biobanks: The HPP will work with biobanking consortia to improve access for proteomics researchers to highly curated and accurately annotated clinical samples collected in a standardized manner.
10. Encourage higher levels of community engagement: The HPP will continue to reach out to the community, supporting findable, accessible, interoperable and reusable (FAIR) data principles^17^, while encouraging and appropriately recognizing all contributions to the re-analysis of proteomics data.

The post SARS-CoV-2 pandemic world will be different. It is likely that new paradigms to accelerate precision medicine will emerge. These will undoubtedly involve global collaboration (even between competing entities) using multidisciplinary approaches that enable the fast-tracking of novel diagnostic tests and precision therapeutics. Almost certainly these outcomes will require knowledge involving the human proteome—celebrated here in the inaugural HPP High-Stringency Blueprint.

## Supplementary information

Supplementary Information
