# Supplementary material for: A high-stringency blueprint of the human proteome
Source: Nat Commun. 2020 Oct 16;11:5301. doi: 10.1038/s41467-020-19045-9 (PMC7568584; doi:10.1038/s41467-020-19045-9)
Supplement: Supplementary file 1 — Supplementary Information [file 41467_2020_19045_MOESM1_ESM.pdf]

Supplementary Information: A High-Stringency Blueprint of the Human Proteome

Human Proteome Project Members

Simon R. Abbey, Subash Adhikari, Ruedi Aebersold[Ø^ꝏØ§], Tiziana Alberto, Ramiro Alonso, Norman L. Anderson, Rolf Apweiler[©§], Seong Beom Ahn, Juan Pablo Albar, Per Andren, Osbaldo R. Antonio, Ron D. Appel, Norie Araki, Alexander Archakov, John Arthur, Stéphane Audebert, Guadalupe Ayala, Mohan Babu, Nicolai Bache, Gary D Bader, Amos Bairoch[§], Mark S. Baker[¤©ꝏ], Kumar Bala, Rosemary Balleine, Nuno Bandeira, Cristina Banfi, Michal Bassani-Sternberg, Ronald C. Beavis, Dörte Becher, Peter Bell, Emøke Bendixen, Laura Beretta, John J.M. Bergeron[©], Frode Berven, Cora N. Betsinger, Rainer Bischoff, Luca Bini, Ruth Birner-Grünberger, Francisco J. Blanco, Jonathon Blackburn, Günter Blobel, Bernd Bodenmiller, Italia Bongarzone, Christoph H. Borchers, Carl Borrebaeck, Ralph A. Bradshaw, Maura Brioschi, Christophe Bruley, Juan J. Calvete, Richard Caprioli, Etienne Caron, Christine Carapito, Elisabet Carlsohn, Steven A. Carr, Alejandro García Carranca, Victor Corasolla Carregari, Paulo Carvalho, Massimo Castagnola, Christine Carapito, Marianna Caterino, Ferdinando Cerciello, John A. Chakel, Subhra Chakraborty, Brian T. Chait, Robert Chalkey, Daniel W. Chan[§ ꝏ], Yu-Sun Chang, Yu-Ju Chen[©], Joel M. Chick, Clizia Chinello, Byung Ok Cho, Je-Yoel Cho, Jin-Young Cho, Sang Yun Cho, Hyewon Chung, Hyung-Min Chung, Maxey C. M. Chung, Federica Ciregia, Robert J. Cotter, Julio Collado-Vides, Stuart Cordwell, Antonella Cormio, Jens Coorssen, Garry Corthals, Fernando J. Corrales[Ø ꝏ], Catherine E. Costello[©], John Cottrell, Juergen Cox, Flora Cozzolino, David Creasy, Ileana M. Cristea[Ø ꝏ], Ben Crossett, Vincenzo Cunsolo, Laura Dagley, Loïc Dayon, Michel Desjardins, Marta del Campo Milan, Eric W. Deutsch[§ꝏ], Umut Dincer, Bruno Domon, Gilberto B. Domont, Will Dracup, Paula Duek, Michael J. Dunn, Ulrich Eckhard, Aled M. Edwards, Sergio Encarnación-Guevara, Jimmy Eng, Fei Fang, Terry Farrah, Mauro Fasano, Thomas E. Fehniger, Catherine Fenselau, David Fenyö, Daniel Figeys, Simona Fontana, Nikolaus Fortelny, Barbara Garavaglia, Benjamin Garcia, Manuel Fuentes Garcia, Jérôme Garin, Alain Gateau, Concha Gil, Jeovanis Gil-Valdes, Laura Giusti, David Goodlett, Angelika Gorg, Susumu Goto, Kun-Liang Guan, Manoj Kumar Gupta, Viviana Greco, Rebekah L Gundry, Tiannan Guo, Amr El Goushy, Harsha Gowda, Rudi Grimm, Lea T. Grinberg, Steven Gygi, Kyudong Han, Samir M. Hanash[©], William S. Hancock[Ø], Yoshitoshi Hirao, Atsushi Hatano, Fuchu He, Qing-Yu He, Joshua Heazlewood, Albert Heck, Henning Hermjakob[§], Magdalena HernándezOrtiz, Michelle Hill, Sophia Hober, Denis Hochstrasser, Leroy E. Hood, Peter Hoffman, Hubert Hondermarck, Daniel Hornburg, Peter Horvatovich, Ruth Huettenhain, Ian Humphery-Smith, Donald F. Hunt, Christie Hunter, Heeyoun Hwang, Federica Iavarone, Vera Ignjatovic, Yasushi Ishihama, Mohammad T. Islam, Mio Iwasaki, Savita Jayaram, Seul-Ki Jeong, Connie Jimenez, Andy Jones, Bill T.W. Jordan, Joshua L. Justice, Jacob Kagan, Eugene Kapp, Takashi Kawamura, Shin Kawano, Neil L. Kelleher, Paul Keown, Jesper Kers, Chae-Yeon Kim, Hoguen Kim, Heui-Soo Kim, HuiSu Kim, Jin Young Kim, Ju-Wan Kim, Yong-In Kim, Chris Kinsinger, Joachim Klose, Daiki Kobayashi, Tadashi Kondo, Markus Kostrzewa, Jeroen Krijgsveld, Ulrike Kusebauch, Martin Kussmann, Bernhard Kuster, Kyung-Hoon Kwon, Joshua LaBaer, Henry Lam, Philipp Lange, Hanno Langen, Irina Larina, Martin R. Larsen, Edward Lau, Thomas Laurell, Pierre Legrain[©], Maggie PY Lam, Lydie Lane[Ø§ꝏ], Albert Lee, Bonghee Lee, Hyoung-Joo Lee, Soo-Yeon Lee, Soo-Youn Lee, Janne Lehtiö, Frédérique Lisacek, Andrey Lisitsa, Danni Li, Kathryn Lilley, Cecilia Lindskog[§ꝏ], Siqi Liu[ꝏ], Joseph Loo, Friedrich Lottspeich, Antonio Lucacchini, Emma K. Lundberg, Michael MacCoss,*

Brendan MacLean, Charlotte Macron, Elisa Maffioli, Fulvio Magni, Alexander Makarov, Parag Mallick, Johan Malm, Mathias Mann, Miguel Marcilla, Katrin Marcus, Federica Marini, Gyorgy Marko-Varga, Daniel Martins-de-Souza, Lennart Martens, Masaki Matsumoto, Rajasree Menon, Helmut E. Meyer, Abidali Mohamedali, Mark P. Molloy, Francesca Monteleone, Maria Monti, Valentina Monti, Orlando Morales-Tarré, Rommel Mathias, Robert L. Moritz[ɸꝏ], Yuki Moriya, David Muddiman, Markus Mueller, Jan Mulder, Clara Musicco, Keun Na, Kazuyuki Nakamura, Edouard C. Nice[§], Eugene Nikolaev, Carol Nilsson, Peter Nilsson, Tommy Nilsson, Toshihide Nishimura, Aleksandra Nita-Lazar, Fabio C.S. Nogueira, Jensen Ole Nørregaard, Antonio NunezGalindo, Shujiro Okuda, Gilbert S. Omenn[¤ꝏ], Christopher M. Overall [Øꝏ], Sandra Orchard, Nicolle H. Packer, Young-Ki Paik[Ø©ꝏ], Vitaliano Pallini, Magnus Palmblad, Giuseppe Palmisano, Akhilesh Pandey, Gun Wook Park, Hyoung-Min Park, Jin Park, Jisook Park, Young-Mok Park, Laurie Parker, Sarah Parker, Scott Patterson, Amanda Paulovich, Stephen R. Pennington[§©ꝏ], Anne Gonzalez de Peredo, Silvia Persichilli, Emanuel F. Petricoin, Giuseppe Petrosillo, Evangelia Petsalaki, Paola Picotti, Luisa Pieroni, Charles Pineau[ꝏ], Peipei Ping, Elena Ponomarenko, Fredrik Pontén, Terence Poon, Vito Porcelli, Kate Poverennaya, Benjamin S. Pullman, Anthony W. Purcell, Xiaohong Qian, Jun Qin, Thierry Rabilloud, Manfred Raida, Sri H. Ramarathinam, Shoba Ranganathan, Roger Reddell, Melinda Rezeli, Kathrin Riedel, Pier Giorgio Righetti, Thibault Robin, Carol Robinson, Phil Robinson, Ana Robles, Karin Rodland, Henry Rodriguez, Michael Roehl, Peter Roepstorff, Hannes Roest, Paola Roncada, Maurizio Ronci, Kate Rosenbloom, Ghasem Hosseini Salekdeh, Rosaria Saletti, Jean-Charles Sanchez, Mathieu Schaeffer, Birgit Schilling, Frank Schmidt, Jan E. Schnitzer, Oliver Schubert, Domitille Schvartz, Jochen M. Schwenk[§], Sarah B. Scruggs, Salvatore Sechi, Richard D. Semba, Samridhi Sharma, Jiahui Shi, Shuichiro Shimada, Albert Sickmann, Richard J. Simpson, Ravi Sirdeshmukh, Richard D. Smith, Michael P. Snyder[©^], Alessio Soggiu, Nestor Solis, Sanjeeva Srivastava, Sudhir Srivastava, Hanno Steen, Peter Stewart, Mark Stolowitz, Naoyuki Sugiyama, Jinshuai Sun, Zhi Sun, Tsuyoshi Tabata, David Tabb, Tomoyo Takami, Naoyuki Taniguchi, W. Andy Tao, Mike Taussig, Gabriella Tedeschi, Hanna Tegel, Charlotte Teunissen, Morten Thaysen-Andersen, Pierre Thibault, Andreas Tholey, Visith Thongboonkerd, Ali Tiss, Tomohiro Uchimoto, Marius Ueffing, Mathias Uhlén[§], Andrea Urbani, Paul J. Utz, Yves Vandenbrouck, Jennifer E. Van Eyk [Øꝏ], Jan van Oostrum , María del Carmen Vargas-Lagunas, Akos Vegvari, Vidya Venkatraman, Olga Vitek, Juan Antonio Vizcaino, James Waddington, Jei Wang, Tong Wang, Xiangdong Wang, Yihao Wang, Yu Watanabe, Susan Weintraub[§ꝏ], Sara Wennersten, Ann Brinkmalm Westman, Mathias Wilhelm, Marc Wilkins, Keith Williams, Melanie White, Bernd Wollscheid, Shujia Wu, Yi Xing, Ping Xu, Keiko Yamamoto, Tadashi Yamamoto, Yoshinori Yamanouchi, Kengo Yanagita, Pengyuan Yang, John Yates III, Jong Shin Yoo, Akiyasu C. Yoshizawa, Shamshad Zarina, Gong Zhang, Hui Zhang, Pumin Zhang, Yao Zhang, Mara Zilocchi and Roman Zubarev.

[©]*Current/Previous/Incumbent HUPO President*
[¤] *Current/Previous HPP Chair*
[ɸ] *Current HPP co-chair*
[^] *Present/Previous HPP Scientific Advisory Board Chair*
[Ø] *Present/Previous C-HPP and B/D-HPP Initiative co-chairs*
[§] *Present/Previous Ab, KB, MS and/or Pathology Resource Pillar co-chairs*
[ꝏ] *Current HPP Executive Committee Member*

Supplementary figure 1

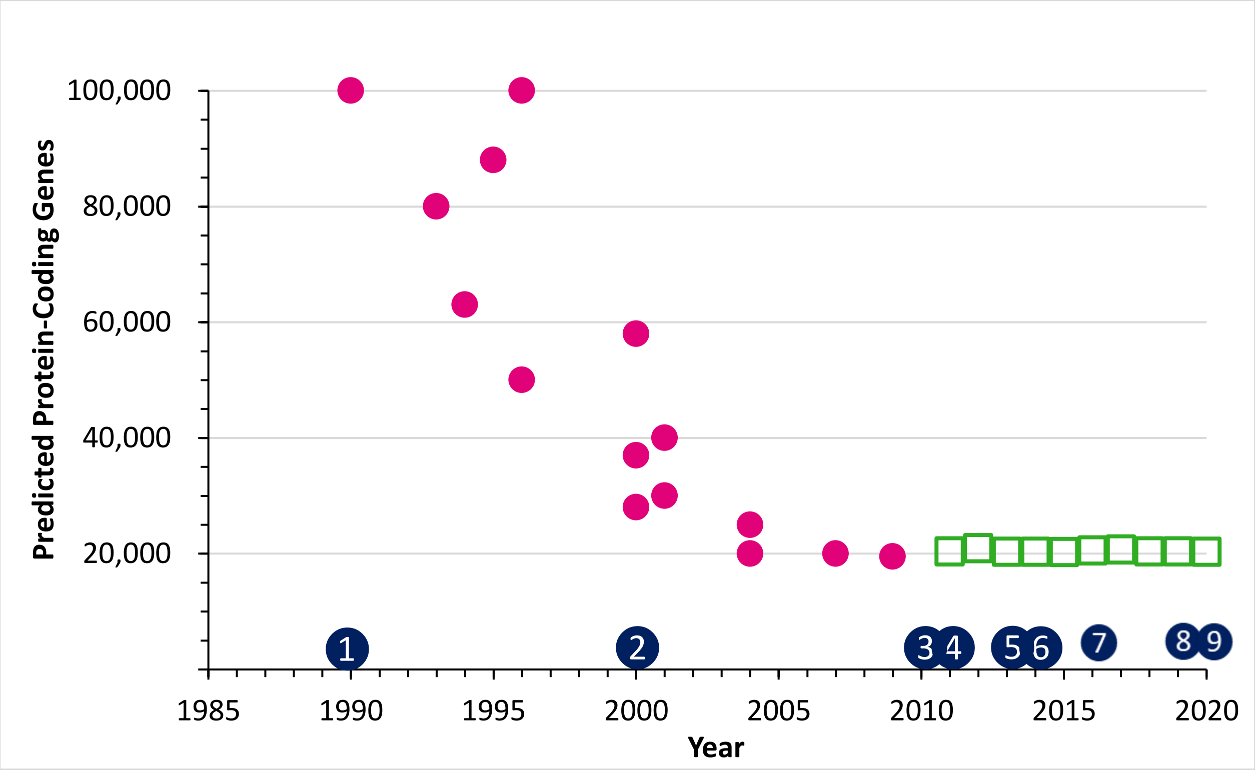

Supplementary Figure 1: *Estimated number of protein-coding genes since HGP Launch.*

*This figure is a composite of data summarised in the Nature News 19th June 2018 article "New human gene tally reignites debate" by S. Willyard that has been updated to capture the 2010-2020 neXtProt PE1+PE2+PE3+PE4+PE5 protein-coding gene cumulative data. X-axis timeline indicates following events; ❶ HGP Launch (1990), ❷ HGP Initial Draft Announced (2000), ❸ HPP Launch (2010), ❹ HUPO partners with neXtProt as HPP Reference KB and neXtProt/HPP agrees to integrate HPA data (2011), ❺ neXtProt/HPP integrates PeptideAtlas data (2013), ❻ neXtProt/HPP integrates SRMAtlas (2014), ❼ HPP MS Data Guidelines v2 released (2016), ❽ HPP MS Data Guidelines v3 released (2019), ❾ neXtProt/HPP integrates MassIVE and HPP High-Stringency Blueprint (2020). Data based upon (●; red closed circle) Willyard, S. Nature News, June 19th, 2018 and (□; **blue open square**) neXtProt 2010-2020 annual HPP PE1,2,3,4,5 data.*
